# Holey graphene frameworks for highly selective post-combustion carbon capture

**DOI:** 10.1038/srep21537

**Published:** 2016-02-16

**Authors:** Shamik Chowdhury, Rajasekhar Balasubramanian

**Affiliations:** 1 Department of Civil & Environmental Engineering, National University of Singapore, 1 Engineering Drive 2, Singapore 117576, Republic of Singapore

## Abstract

Atmospheric CO_2_ concentrations continue to rise rapidly in response to increased combustion of fossil fuels, contributing to global climate change. In order to mitigate the effects of global warming, development of new materials for cost-effective and energy-efficient CO_2_ capture is critically important. Graphene-based porous materials are an emerging class of solid adsorbents for selectively removing CO_2_ from flue gases. Herein, we report a simple and scalable approach to produce three-dimensional holey graphene frameworks with tunable porosity and pore geometry, and demonstrate their application as high-performance CO_2_ adsorbents. These holey graphene macrostructures exhibit a significantly improved specific surface area and pore volume compared to their pristine counterparts, and can be effectively used in post-combustion CO_2_ adsorption systems because of their intrinsic hydrophobicity together with good gravimetric storage capacities, rapid removal capabilities, superior cycling stabilities, and moderate initial isosteric heats. In addition, an exceptionally high CO_2_ over N_2_ selectivity can be achieved under conditions relevant to capture from the dry exhaust gas stream of a coal burning power plant, suggesting the possibility of recovering highly pure CO_2_ for long-term sequestration and/or utilization for downstream applications.

The global annual mean concentration of CO_2_ in the atmosphere has increased markedly from pre-industrial levels of about 280 parts per million (ppm) to 400 ppm in 2013^1^. Further increases are expected as the annual burning of fossil fuels is likely to continue to rise rapidly to meet the spiraling energy demand of an escalating human population. A massive effort must be undertaken to reduce the amount of CO_2_ entering the atmosphere because of its contribution to global climate change^2^. There is a widespread consensus that the development and deployment of a broad portfolio of advanced energy technologies would be the most effective and sustainable approach to bring about stabilization of the atmospheric CO_2_ concentrations^1^. While energy efficiency improvements and increased use of renewable energy resources are a long-term proposition of this portfolio^3^, carbon capture, utilization and storage (CCUS) is a short to medium term technological option for mitigating anthropogenic energy-related CO_2_ emissions^4^,^5^,^6^. Of the various strategies (i.e., pre-combustion, post-combustion and oxy-fuel combustion) and numerous technologies (e.g., absorption, adsorption, membrane separation and cryogenic distillation) that are currently being explored to capture CO_2_ from fossil-fuelled power plants and other large industrial sources^1^,^7^, post-combustion capture using porous adsorbents constitutes a promising solution because of its simplicity and cost effeciency^8^. A variety of porous solids have been extensively investigated among which porous carbons are particularly well-suited to be deployed within post-combustion CO_2_ capture systems, owing to their abundant porosity and ease of fabrication^8^.

Three-dimensional (3D) graphene-based frameworks (3D-GFs) such as aerogels, hydrogels, foams, sponges, and nanomesh are an important new class of porous carbon materials, attracting increasing attention for energy-related applications^9^,^10^. Due to their unique continuously interconnected networks, 3D-GFs exhibit large accessible surface area, high pore volume, excellent stability, good flexibility, and adequate mechanical strength^11^,^12^. As such, these materials can also serve as robust adsorbents for capturing CO_2_ emanating from the combustion of fossil fuels. For example, a graphene hydrogel, made from the self-assembly of graphene oxide sheets *via* a hydrothermal process, had a 3D porous structure, high specific surface area (530 m^2^ g^−1^) and large pore volume (0.66 cm^3^ g^−1^), and consequently showed great promise for CO_2_ adsorption and separation applications^13^. However, such 3D-GFs generally lack well-defined mesopores and/or micropores^9^, which can substantially limit the efficiency of mass transport and gas diffusion through the pore networks. Therefore, it is highly desirable to develop hierarchical porous 3D-GFs by integrating holey graphene nanosheets into a macroscopic 3D interconnected network structure.

Here, we describe a facile and scalable approach to produce holey graphene frameworks (HGFs) by etching in-plane nanopores into graphene sheets through a wet chemical method, that allows a tunable pore geometry and controllable pore density, followed by their self-assembly to form a 3D interconnected porous macrostructure. The as-synthesized HGFs were found to have high specific surface area and large pore volume with well-developed mesoporosity. We further show that these HGFs are extremely attractive for post-combustion CO_2_ adsorption applications, including good gravimetric capacity, rapid removal capability, superior cycling stability, and moderate initial isosteric heat of adsorption. Most importantly, the CO_2_ over N_2_ adsorption selectivity is among the highest at operating conditions pertinent to post-combustion capture from coal-fired power plants, which would indeed be extremely beneficial for extracting a high-purity CO_2_ stream from flue gases for deep underground storage or other industrial applications.

## Results

### Preparation and characterization of HGFs

**Figure 1:**
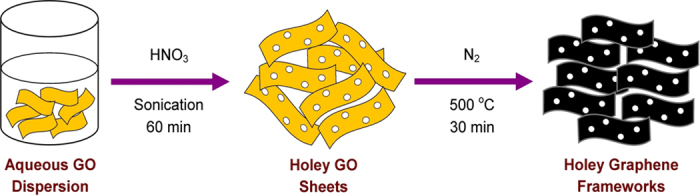
Illustration of the major steps involved in the preparation of HGFs. The synthesis involves etching of in-plane nanopores into GO sheets and their self-assembly into a 3D interconnected network structure.

Figure 1 presents a schematic of the methodology adopted for preparing HGFs. A homogenous aqueous dispersion of graphene oxide (GO) was mixed with a desired amount of 69 wt.% concentrated HNO_3_ under stirring. The mixture was then subjected to sonication in a water bath at sufficient acoustic pressure and room temperature for 1 h. After removing the residual HNO_3_ by centrifuging and washing the reaction mixture, the as-obtained holey graphene oxide (HGO) was annealed under N_2_ atmosphere at 500 °C for 30 min in a horizontal tube furnace. During the thermal annealing process, the HGO sheets were reduced and self-assembled to form 3D HGF. Three different solutions of increasing HNO_3_ concentrations were tested, corresponding to a GO suspension/HNO_3_ volume ratio of 1/3 (mL/mL) (I), 1/6 (mL/mL) (II) and 1/9 (mL/mL) (III). The resulting HGF were labeled as HGF-I, HGF-II and HGF-III, respectively. For comparison, we also synthesized non-holey GFs (NGFs) using the same procedure but without any acid treatment. Compared to the template-assisted chemical vapor deposition (CVD) method, the current synthesis method is simpler and economically more attractive, making the process readily scalable for large scale production of porous graphene materials.

**Figure 2:**
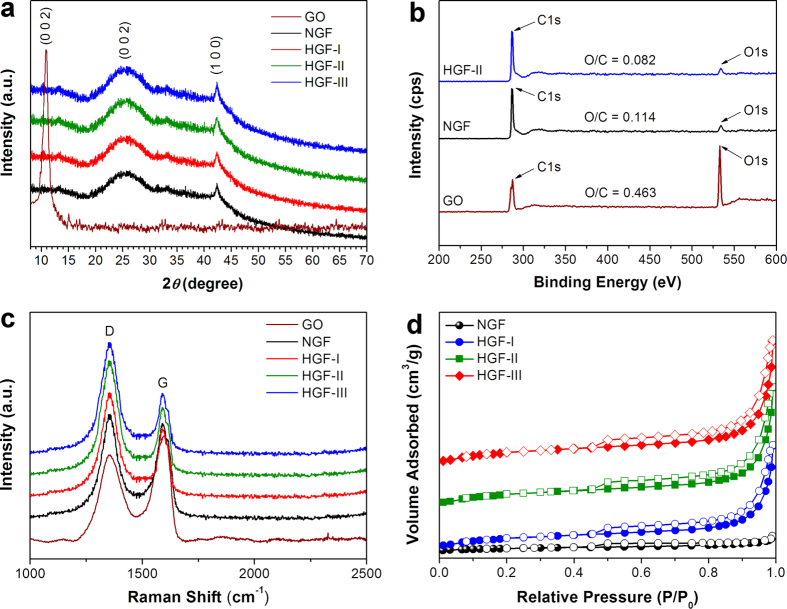
Structural characterization of HGFs. (**a**) Wide angle XRD patterns, (**b**) XPS survey spectra, (**c**) Raman spectra and (**d**) N_2_ adsorption/desorption isotherms of GO, NGF, and HGFs. The solid and open symbols represent adsorption and desorption, respectively.

The wide-angle X-ray diffraction (XRD) patterns of all the HGFs were very similar to those of typical *sp*^2^-bonded carbons, exhibiting the development of crystalline graphene structure (Fig. 2a). The characteristic GO peak at 2*θ* = 11° was absent in all the samples. Instead, two strong and broad peaks were observed at around 2*θ* = 25° and 43.3°, conforming to the graphitic (0 0 2) and (1 0 0) crystal planes, respectively. In accordance with the XRD results, the X-ray photoelectron spectroscopy (XPS) survey scan of the HGFs indicated that GO was sufficiently reduced by thermal annealing at 500 °C, with a significant deoxygenation during the acid etching process (Fig. 2b) (see Supplementary Fig. 1 for the XPS survey scans of HGOs). The reduction of oxygenated functional groups was also confirmed by Fourier transform infrared (FTIR) spectroscopy, as shown in Supplementary Fig. 2.

Further structural information about the prepared HGFs was obtained from Raman spectroscopy. All the samples without exception showed three prominent absorption bands at about 1350 and 1596 cm^−1^, which correspond to the well-documented D (related to defect in *sp*^2^ lattice) and G (related to pristine *sp*^2^ graphitic layer) bands, respectively (Fig. 2c)^14^,^15^,^16^. The D/G intensity ratios of the HGFs (*I*_D_/*I*_G_ = 1.09 for HGF-I, 1.20 for HGF-II, and 1.26 for HGF-III) were found to be larger than that of GO (*I*_D_/*I*_G_ = 0.77), and are in agreement with the literature^17^. In contrast to the Raman spectrum of NGF (*I*_D_/*I*_G_ = 1.02) obtained without acid sonication, the remarkably strong D band intensity for the HGF samples could be ascribed to the introduction of in-plane carbon vacancy defects (pores) during the etching of GO with HNO_3_. Because the *sp*^3^ carbon atoms are chemically more active than the *sp*^2^ carbons in the bulk of GO^18^,^19^, HNO_3_ molecules mainly attack the oxygenic defect regions, leading to the preferential removal of oxygenated carbon atoms and generation of carbon vacancies that gradually extend into nanopores^19^. The role of oxygenated carbon species in the formation and evolution of pores is corroborated by the large difference between the O/C atomic ratios of GO (atomic O/C = 0.46) and HGOs (atomic O/C = 0.32, 0.24 and 0.17 for HGO-I, HGO-II and HGO-III, respectively) calculated from their XPS survey scans (Fig. 2b and Supplementary Fig. 1). Additionally, as the defective carbon sites are usually distributed throughout the basal plane of GO^20^, the etching process could occur across the entire graphene sheets to result in abundant in-plane pores of a few nanometres all over the sheet^21^. However, excessive HNO_3_ would lead to a more aggressive etching, enlarging the pore size of holey graphene, as inferred from the increase in the *I*_D_/*I*_G_ ratio with increasing etchant concentration.

**Figure 3:**
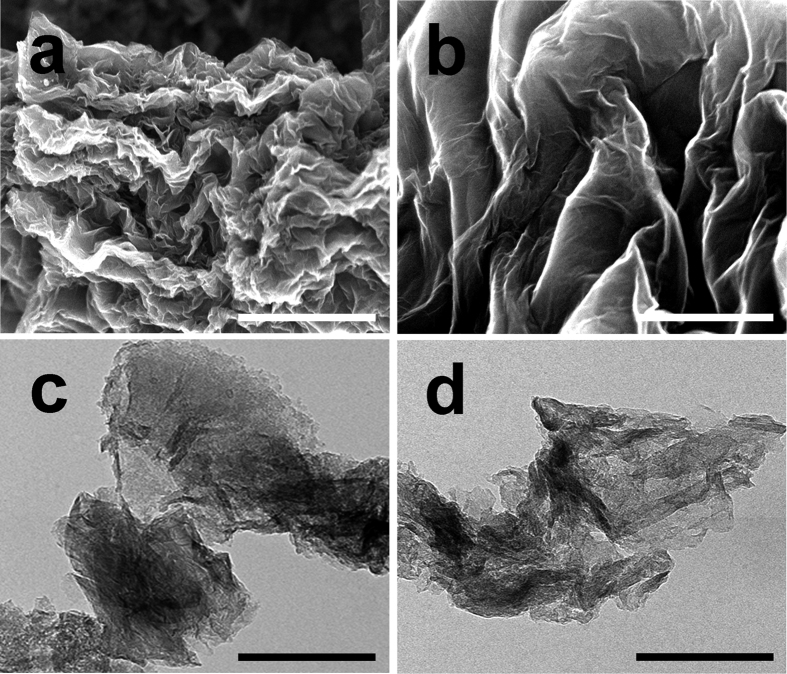
Morphological characterization of HGFs. FESEM images of (**a**) HGF-II and (**b**) NGF. (**c,d**) TEM images of HGF-II. The scale bars in (**a–b**), (**c**) and (**d**) represent 1 μm, 100 nm and 50 nm, respectively.

The porous morphology of the samples was confirmed by field emission scanning electron microscopy (FESEM) and transmission electron microscopy (TEM). From the FESEM micrographs of the HGFs (Fig. 3a and Supplementary Fig. 3), a distinct 3D porous framework consisting of interconnected networks of randomly oriented sheet-like structures was clearly observed. These sheets were rather thin and wrinkled as revealed by low-magnification TEM (Fig. 3c,d), indicating the efficient self-assembling of 2D porous graphene nanosheets into 3D macrostructures through a combination of hydrophobic and π–π interactions^22^,^23^. The NGF, on the other hand, displayed a planar but contorted structure (Fig. 3b), ascertaining the involvement of HNO_3_ molecules in pore development, and thus the change in morphological appearance of the graphene layers.

To assess the pore structures of the developed graphene frameworks, the N_2_ adsorption/desorption isotherms were measured. All the samples generated a type IV isotherm with a type H3 hysteresis loop in the relative pressure region of 0.45–1.0 (Fig. 2d), often associated with non-rigid aggregates of plate-like particles forming slit-shaped mesopores^24^. The calculated Brunauer–Emmett–Teller (BET) specific surface areas and the total pore volumes of the as-prepared HGFs were remarkably higher than that of NGF (Supplementary Table 1). The HGF-III sample displayed the largest BET surface area (524 m^2^ g^−1^) and total pore volume (1.27 cm^3^ g^−1^), which is likely due to the creation of greater porosity in the bulk sample from excessive etching at higher acid concentrations. Although the specific surface areas of our developed HGFs are substantially lower than the theoretical surface area of 2630 m^2^ g^−1^ of an individual graphene sheet^25^, they are still comparable to, or greater than those of graphene aerogel (512 m^2^ g^−1^)^26^, graphene sponge (418 m^2^ g^−1^)^27^, and graphene nanoplates (480 m^2^ g^−1^)^28^. The pore size distributions (PSDs) obtained using the Barrett–Joyner–Halenda (BJH) method revealed that most of the pore volume was contributed by pores of diameter less than 10 nm (Supplementary Fig. 4), with a more prominent pore size distribution in the range of 3–4 nm, implying the presence of narrow mesopores in the basal plane of all the HGFs. While the surface area and total pore volume of the HGFs increased considerably on increasing the volume of acid to the GO precursor, the BJH pore size decreased on increasing the GO/HNO_3_ ratio from 1/3 to 1/6 (Supplementary Table 1), leading to a more compact structure for HGF-II. However, further increase in HNO_3_ levels yielded HGFs with larger holes, as was evident from the shift of the PSD maxima from 3.29 nm for HGF-II to 3.74 nm for HGF-III. This finding is also consistent with the variations in the *I*_D_*/I*_G_ ratio of the samples, suggesting that the degree of etching and the concomitant porosity of the frameworks can be conveniently tuned by the proportion of GO to HNO_3_.

Wettability of the HGFs was also quantified by measuring their water contact angles (Supplementary Fig. 5). Small contact angles (<90°) correspond to high surface wettability (hydrophilic), while large contact angles (>90°) correspond to low surface wettability (hydrophobic)^29^. As can be seen from Supplementary Fig. 5, the HGFs exhibited poor wetting with water contact angle greater than 130°. The strong hydrophobicity can be attributed to the surface roughness induced by the morphology of HGFs.

### CO_2_ adsorption performance

**Figure 4:**
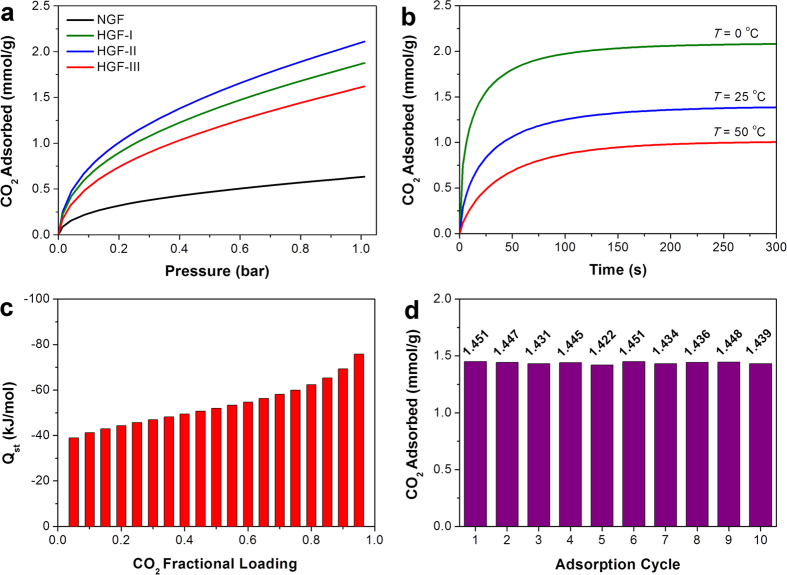
CO_2_ adsorption performance of HGFs. (**a**) Pure component CO_2_ adsorption isotherms of NGF and HGFs at standard temperature and pressure (0 °C and 1 bar). (**b**) CO_2_ adsorption kinetics of HGF-II at different temperatures. (**c**) Calculated isosteric heat of adsorption for HGF-II as function of CO_2_ loading. (**d**) Cyclic CO_2_ adsorption performance of HGF-II at 25 °C.

The efficacy of the synthesized HGF samples for post-combustion CO_2_ capture applications was evaluated by measuring their low-pressure CO_2_ adsorption capacity using a volumetric gas adsorption apparatus. Under identical experimental conditions, the HGFs exhibited considerably improved CO_2_ adsorption in comparison with their non-holey counterpart (Fig. 4a). The CO_2_ adsorption capacity of the former (*ca.* 2.11 mmol g^−1^) was about 3.3 times that of the latter (*ca.* 0.63 mmol g^−1^) due to the presence of small slit-pores. The adsorption of some amount of CO_2_ on NGF could be attributed to the voids between the graphene sheets, generated by the layer-by-layer self-assembly during thermal annealing. In addition, a steep rise in the adsorption capacity at pressure <0.2 bar was found for all the HGFs. The interconnected platelet structure with small mesopores caused more efficient CO_2_ diffusion and provided enough space to avoid the steric hindrance effect^30^. As a result, the entire volume of pores in HGFs was readily accessible. Furthermore, no distinct plateau was noticed in the isotherms for the pressure range investigated, indicating that the samples can adsorb greater volume of CO_2_ at higher pressure. However, the CO_2_ adsorption capacity did not show any apparent correlation with the specific surface area, or pore volume of the HGFs. Notably, the equilibrium CO_2_ uptake at 0 °C and 1 bar increased in the following order: HGF-I (1.62 mmol g^−1^) <HGF-III (1.87 mmol g^−1^) <HGF-II (2.11 mmol g^−1^). Even though HGF-III showed the highest BET surface area and total pore volume, the HGF-II material gave the best performance probably because of its smallest mesopore size among all the samples. The contribution of smaller pores in low-pressure CO_2_ adsorption is also well-documented in the literature^31^. Moreover, the CO_2_ adsorption in HGF-II is better than or comparable to other graphene-based materials at similar temperature and pressure conditions (Supplementary Table 2). Therefore, further CO_2_ adsorption studies under flue-gas-like conditions were conducted using the HGF-II sample (Supplementary Fig. 6). A CO_2_ partial pressure of 0.15 bar was considered as the representative value for post-combustion carbon capture from flue gas^7^. When adsorption temperature was increased from 0 °C to 25 °C, the amounts of CO_2_ adsorbed on HGF-II decreased by 41% at 0.15 bar (0.91 mmol g^−1^ at 0 °C vs. 0.53 mmol g^−1^ at 25 °C), and 34% at 1 bar (2.11 mmol g^−1^ at 0 °C vs. 1.40 mmol g^−1^ at 25 °C). This uptake is, however, higher than the value of 0.37 mmol g^−1^ at 0.15 bar and 30 °C obtained for zeolite^32^, 0.11 mmol g^−1^ at 0.15 bar and 25 °C measured for a metal organic framework (MOF)^33^, and is comparable to the recently reported value of ~0.55 mmol g^−1^ for activated carbons at 0.15 bar and 25 °C^34^. Further reduction in CO_2_ uptake at 50 °C is likely associated with the exothermicity of the adsorption process. Nevertheless, the adsorbed amounts of CO_2_ still remain to be 0.34 mmol g^−1^ and 1.02 mmol g^−1^ at 0.15 bar and 1 bar, respectively. Additionally, there was no distinct hysteresis between the CO_2_ adsorption and desorption branches (Supplementary Fig. 7). This observation supports the conclusion that HGF-II could be potentially applicable for capturing CO_2_ from coal-fired power plant emissions, because near-complete regeneration of the fairly good amount of CO_2_ adsorbed was possible for subsequent sequestration or utilization.

The measured pure component isotherm data for CO_2_ on HGF-II were fitted with the Toth model^35^ to elucidate the underlying adsorption mechanism. The fitted isotherm constants are listed in Supplementary Table 3 while the Supplementary Fig. 8 presents a comparison of CO_2_ loadings in HGF-II with the isotherm fits. The goodness-of-fit of the Toth model over the entire pressure and temperature range indicates that CO_2_ molecules were adsorbed on HGF-II in multimolecular layers because of the high degree of adsorbent surface heterogeneity. This implies that the adsorption space can accommodate more than one layer of molecules and not all adsorbed molecules are in contact with the surface layer of the adsorbent^36^. Some type of lateral interactions also takes place between the adsorbed molecules^37^. Moreover, the heat evolved during adsorption (i.e., heat of adsorption) is of the same order of magnitude as physical adsorption (5–40 kJ mol^−1^)^38^, suggesting that CO_2_ was strongly physi-sorbed onto HGF-II essentially through van der Waals forces (also known as dispersion-repulsion forces) and electrostatic forces (also known as Coulombic interactions), arising mainly from quadrupole-quadrupole interactions between CO_2_ and the defective graphene surface^39^.

Since a typical adsorption cycle in a large-scale commercial CO_2_ capture facility will likely be on the order of minutes, the consideration of the rate of adsorption is also very important when evaluating the performance of any new solid CO_2_ adsorbent^40^. Indeed, a rapid adsorption will ensure fast cycling times, thereby reducing equipment size and allowing for efficient utilization of the adsorbent^41^. Figure 4b presents the kinetic curves for the HGF-II material at 0, 25 and 50 °C. It can be seen that CO_2_ uptake occurred at high adsorption rates, with more than 95% of CO_2_ being adsorbed within 3 min, over the range of temperature investigated. The potentially fast adsorption was the outcome of fewer diffusion limitations owing to the narrow mesoporosity of the sample. These results clearly indicate that HGF-II can effectively separate CO_2_ from flue gases while operating with very short adsorption/desorption cycle times, which would in turn be economically advantageous for commercial deployment.

As flue gas streams emanating from post-combustion coal-fired power plants contain relatively low concentrations of CO_2_ (15%) and large quantities of N_2_ (75%)^42^, a potential CO_2_ capture adsorbent must also be capable of selectively adsorbing the CO_2_ component of the gas mixture, such that only pure CO_2_ is captured and subjected to sequestration^43^. In fact, the purity of the recovered CO_2_ has a significant impact on the technical feasibility of developing an adequate transport and storage infrastructure^44^ and is, therefore, critical to CCUS economics. Thus, in this study, the single-component adsorption isotherm for N_2_ was measured and compared with the experimental CO_2_ isotherm data to evaluate the adsorption selectivity for CO_2_ over N_2_ (Supplementary Fig. 9). On a gravimetric basis, HGF-II could adsorb about 29 times more CO_2_ than N_2_ at 25 °C and 1 bar. The high selectivity is a consequence of the exceptionally large polarizability and quadrupole moment of CO_2_ (29.11 × 10^−25^ cm^−3^ and 4.30 × 10^−26^ esu^−1^ cm^−1^, respectively) than N_2_ (17.40 × 10^−25^ cm^−3^ and 1.52 × 10^−26^ esu^−1^ cm^−1^, respectively)^45^. Because the combustion of coal in air generates flue gas with a total pressure of approximately 1 bar, the selectivity calculation for CO_2_ over N_2_ is best performed using the adsorption capacities at pressures of approximately 0.15 bar for CO_2_ and 0.75 bar for N_2_ as follows^43^:


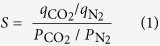
where *S* is the selectivity factor, and, 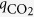 and 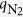 are the amount of CO_2_ and N_2_ adsorbed at their respective equilibrium partial pressures 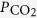 and 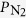. The purity of the captured CO_2_ can be then determined from the expression^46^:


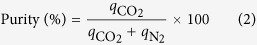
Attractively, HGF-II exhibited an excellent flue gas normalized CO_2_/N_2_ selectivity of 70, suggesting that high-purity CO_2_ (93.34%) could be recovered from coal-fired dry flue gas. This would in turn enable economic compression, transportation and storage/utilization, and also reduce the costs of installing and operating CCUS systems at power plants. Noticeably, the CO_2_/N_2_ selectivity as well as the purity of the captured CO_2_ for HGF-II is considerably higher than that of several well-known porous solid CO_2_ adsorbents (Supplementary Table 4), particularly activated carbons, at pressures and temperatures relevant to practical post-combustion carbon capture. These findings further highlight the suitability of HGF-II as an excellent alternative for the efficient recovery of CO_2_ from flue gases emitted by coal-burning power stations.

Quantification of the isosteric heat of adsorption (*Q*_st_) is also important for the practical design of an adsorption-based CO_2_ capture unit. It governs the local changes in the adsorbent temperature inside an adsorption column during the adsorption/desorption process, which in turn influences the local adsorption equilibria and kinetics, ultimately dictating the overall gas separation efficiency^47^. A moderately high *Q*_st_ ensures both efficient CO_2_ capture and subsequent facile desorption to regenerate the adsorbents^42^. In the present study, the isosteric heat of adsorption of pure CO_2_ on HGF-II was determined from the van’t Hoff equation by using the temperature-dependent Toth isotherm parameters^48^. At the limit of zero coverage, the isosteric heat of adsorption corresponds to the molar enthalpy of adsorption. This initial *Q*_st_ of –30.78 kJ mol^−1^ compares well with the previously reported data for pillared graphene frameworks^49^, and is lower than those of zeolites^50^,^51^, activated carbons^52^,^53^, and MOFs^54^,^55^. Additionally, the zero-coverage *Q*_st_ value at the borderline of strong physisorption and weak chemisorption (*ca.* 25–50 kJ mol^−1^)^56^ is likely to afford a lower energy penalty for regenerating the spent HGF-II adsorbent, which would be of potential benefit for reducing the total energy requirements of post-combustion CO_2_ capture. Figure 4c illustrates the variation of *Q*_st_ as a function of the amount of CO_2_ adsorbed for the HGF-II adsorbent. Significant increase in the isosteric heat was observed, particularly at higher CO_2_ loadings, reaching a maximum of about –75.89 kJ mol^−1^ close to saturation. This behavior is due to pronounced lateral interactions between the adsorbed CO_2_ molecules, as was verified by calculating the interaction energy between the adsorbed CO_2_ molecules from the Fowler–Guggenheim model^57^. The interaction energy, turned out to be positive (7.33 kJ mol^−1^), confirming the existence of attractive intermolecular forces among the adsorbed quadrupolar CO_2_ molecules. Hence, with more CO_2_ molecules present at higher surface loadings, the *Q*_st_ values were higher than at lower surface loadings. Similar dependence of the isosteric heats of adsorption on surface coverage was also found in other recent works on CO_2_ adsorption by carbonaceous materials^58^,^59^,^60^.

To further investigate the practical utility and reliability of HGF-II for post-combustion CO_2_ capture applications, cyclic CO_2_ adsorption/regeneration experiments were conducted by swinging the pressure between vacuum (<0.01 bar) and 1 bar at 25 °C. As shown in Fig. 4d, the amount of CO_2_ uptake by HGF-II remained virtually constant with no deterioration in the adsorption capacity even after ten adsorption/regeneration cycles. In addition, the regenerated HGF-II produced similar bands in the FTIR spectrum to those previously observed before the adsorption measurements (Supplementary Fig. 10). It, therefore, appears that the adsorbed CO_2_ molecules could be effectively desorbed without destroying the intrinsic structure of the adsorbent during the regeneration step, displaying the stability of our developed HGF-II material for prolonged cyclic operation in CCUS units.

## Discussion

We have successfully demonstrated a simple and scalable method to prepare hydrophobic HGFs with superior CO_2_ adsorption properties, through acid etching of GO followed by its thermal annealing and self-assembly into 3D interconnected network structure. Our developed HGF materials have several unique features to deliver a high-performance in CO_2_ capture applications. First, the graphene sheets in our HGFs are highly crumpled and interconnected to prevent them from face-to-face aggregation and to maintain a highly porous 3D network structure, hence providing a large accessible surface area (497 m^2^ g^−1^) and more “space” (1.22 cm^3^ g^−1^) for adsorbing and storing CO_2_. Second, the nanopores in the HGFs have dimensions in the narrow mesopore range, indicating a low-resistant pathway for the diffusion of CO_2_ molecules in the frameworks. Last, the HGFs are extremely hydrophobic (water contact angle >130°) due to their surface roughness and porosity, thus avoiding the co-adsorption of moisture while retaining a high adsorption capacity for CO_2_ under flue gas conditions. This also represents a conspicuous improvement over zeolites, which are essentially hydrophilic, as well as MOFs, in which the metal–ligand bond is susceptible to hydrolysis and can lead to the collapse of the framework structure upon contact with moist flue gas^43^. Consequently, our HGFs maintained a maximum adsorption uptake of up to 2.11 mmol g^−1^ at 1 bar of dry CO_2_. While recent research studies on CO_2_ adsorption have focused extensively on achieving ever higher equilibrium adsorption capacities with little or no attention to adsorption kinetics^61^, we have investigated the rate of CO_2_ uptake in detail. This is because a high equilibrium uptake does not necessarily translate to a better cyclic adsorption performance as it leads to a longer breakthrough time^61^, which in turn implies a smaller number of cycles for the same gas throughput. Such a possibility arises because switching from adsorption to desorption is carried out when the outlet concentration increases to a certain fraction of the feed concentration^61^. Therefore, although some zeolites and many MOFs exhibit higher CO_2_ adsorption capacity than our developed HGF adsorbent, their long breakthrough time is likely to result in extended adsorption periods as well as a reduction of the operating efficiency, ultimately compromising the overall productivity and economics of the CO_2_ capture process. In contrast, the moderate CO_2_ capture capacity of our HGFs together with their rapid kinetics suggests that CO_2_ can be effectively separated from flue gas streams while operating with short adsorption cycle times. This attribute would indeed be beneficial for practical industrial applications. Moreover, the adsorption capacity and the structural integrity of HGFs were preserved through multiple adsorption/desorption cycles, demonstrating the stability of these holey graphene macrostructures for long-term cyclic operation. Furthermore, our comparison of CO_2_ over N_2_ selectivity for various adsorbents under conditions representative of those encountered in coal-fired power plants, reveals the exceptionally superior performance of our HGFs in removing CO_2_ from post-combustion flue gas mixtures. The desorbed CO_2_ concentration could reach above 93%, which is economically advantageous for subsequent utilization as a feedstock in the chemical industry or permanent storage in deep underground geological formations. Most importantly, the energy input required to regenerate the spent HGFs is low as inferred from the moderate initial isosteric heat of –30.78 kJ mol^−1^, reflecting an energy-efficient CO_2_ adsorbent. Nevertheless, flue gases from power plants also contain other trace combustion by-products (such as CO, NO_X_, and SO_X_) which may affect the CO_2_/N_2_ separation performance through competitive adsorption. Hence, further investigations need to be conducted with simulated flue gas mixtures for fully evaluating the effectiveness of our developed HGFs for deployment within real-world CO_2_ capture systems.

## Methods

### Materials

Graphite powder (<20 μm) was purchased from Sigma-Aldrich and used as received. Sulfuric acid (H_2_SO_4_, 98 wt.%, Merck), nitric acid (HNO_3_, 69 wt.%, Honeywell), phosphoric acid (H_3_PO_4_, 85 wt.%, J.T. Baker), potassium permanganate (KMnO_4_, Acros Organics), hydrogen peroxide (H_2_O_2_, 30 wt.%, Sigma-Aldrich), and hydrazine hydrate (N_2_H_4_, 50–60 wt.%, Sigma-Aldrich) were used as available from the supplier without any further purification.

### Synthesis of GO

GO was prepared from natural graphite powder by an improved Hummer’s method. In brief, a 9:1 mixture of concentrated H_2_SO_4_/H_3_PO_4_ (360:40 mL) was added to a mixture of graphite powder (3 g, 1 wt. equiv.) and KMnO_4_ (18 g, 6 wt. equiv.), producing a slight exotherm to 35–40 °C. The reaction mixture was further heated to 50 °C and stirred for 12 h. It was then cooled to room temperature and poured onto ice (~400 mL) with 3 mL H_2_O_2_. Finally, the mixture was centrifuged and the supernatant was decanted away. The remaining solid material was rinsed repeatedly with deionized water until the pH of the solution was neutral. After filtration and drying in air at room temperature, GO was obtained.

### Synthesis of HGO

HGO was synthesized through chemical etching of GO with HNO_3_. Typically, a weighed amount of GO was suspended in deionized water (10 mL). The resulting inhomogeneous yellow-brown dispersion was then vigorously stirred until a homogeneous solution was obtained. To this dispersion, a desired amount of 69 wt.% concentrated HNO_3_ was added under stirring. The mixture was then sonicated in an Elmasonic S 60 H ultrasonic bath (550 W, 37 kHz) (Elma Schmidbauer GmbH, Germany) at room temperature for 1 h. Following sonication, the mixture was allowed to settle at room temperature for an hour, after which the solid was recovered by centrifugation, repeatedly washed with deionized water to neutrality, and finally dried in air. Three different concentrations of HNO_3_ were tested, corresponding to a GO suspension/HNO_3_ volume ratio of 1/3 (mL/mL) (I), 1/6 (mL/mL) (II) and 1/9 (mL/mL) (III). Accordingly, the resulting materials were labeled as HGO-I, HGO-II and HGO-III, respectively.

### Synthesis of HGFs

HGFs were prepared according to the following procedure. The as-prepared HGO was first loaded on a ceramic boat and placed at the center of a horizontal tube furnace (TMH12, Elite Thermal Systems Ltd., UK). The system was then purged with N_2_ (500 mL min^−1^) for 10 min to flush out the air in the tube. After that, the sample was annealed at 500 °C (with a ramp rate of 5 °C min^−1^) under the N_2_ atmosphere for 30 min. The sample was then left to cool naturally to room temperature in the furnace. Depending on the HGO precursor, the materials thus obtained were denoted as HGF-I, HGF-II, and HGF-III, respectively. For comparison, NGFs were synthesized from GO using the same procedure but without any acid treatment.

### Characterization

Wide angle XRD patterns were recorded on a Bruker D8 ADVANCE (Bruker Co., Germany) X-ray diffractometer equipped with Ni-filtered Cu Kα radiation (λ = 0.15 nm) operating at 40 kV and 40 mA. XPS data were acquired using a VG ESCA 220i-XL imaging system (Thermo VG Scientific Ltd., UK). Monochromatic Al Kα X-ray (hν = 1486 eV) was employed for analysis with a photoelectron take-off angle of 90° to the surface plane. The analysis area was approximately 700 mm in diameter while the maximum analysis depth was in the range of 4–8 nm. FTIR spectra were collected on a Varian Excalibur 3100 FTIR spectrometer (Varian Inc., USA) with a spectral resolution of 2 cm^−1^. Raman spectra were recorded on a RM 2000 microscopic confocal Raman spectrometer (Renishaw PLC, UK) using a 514 nm laser beam. FESEM was performed on a JEOL JSM-6700F (JEOL Ltd., Japan) field emission microscope operated at an electron accelerating voltage of 15 kV. The samples were mounted on an aluminium stub with carbon adhesive tape and coated with a thin layer of platinum under high vacuum (10^−3^–10^−7^ Mbar) conditions using a Hitachi E-1030 ion sputter (Hitachi Co. Ltd., Japan) before the FESEM analysis. TEM was carried out on a JEOL JEM 2010F (JEOL Ltd., Japan) transmission electron microscope operated at 200 keV. For TEM measurements, the samples were ultrasonicated in ethanol to form a homogeneous suspension, dropped on a 200 mesh copper TEM grid coated with a thin amorphous carbon film, and then allowed to dry in air. The textural characteristics were quantified by measuring the N_2_ adsorption/desorption isotherms at –196 °C in a Micromeritics ASAP 2020 surface area and porosity analyzer (Micromeritics Instrument Co., USA). All samples were outgassed at 150 °C under vacuum for 1 h prior to the N_2_ adsorption measurements. Dynamic water contact angle measurements were performed at room temperature (23 °C) with deionized water using a contact angle measurement setup equipped with a camera (VCA optima, AST Products Inc., USA). Before the measurements, all the samples were dried at 120 °C for 24 h. The dried samples were then pressed between two glass slides that had been previously rinsed with absolute ethanol and dried with a stream of N_2_ gas. After removing the upper slide, the exposed sample surface was used for conducting the contact angle measurements. The reported contact angle for each sample is the average of at least three independent measurements.

### Gas adsorption measurements

CO_2_ adsorption equilibria of the as-prepared materials were measured volumetrically in a Micromeritics ASAP 2020 adsorption apparatus (Micromeritics Instrument Co., USA) at three different temperatures (0, 25 and 50 °C) and pressures of up to 1 bar. The adsorption temperature was controlled by using a Dewar bottle with a circulating jacket connected to a thermostatic bath utilizing water as the coolant. About 100 mg of adsorbent sample was used for the adsorption studies. Before each adsorption experiment, all the samples were degassed at 150 °C under vacuum for 1 h to desorb any moisture and organics. The CO_2_ adsorption kinetics (adsorption amount as a function of time) was also measured in the Micromeritics ASAP 2020 system using a built-in function (“Rate of Adsorption”) at the same time when the adsorption equilibrium data were collected. The change in gas pressure and adsorption volume with time, after the CO_2_–reservoir was connected to the sample chamber, was first registered and then converted into transient adsorption uptakes to generate the adsorption kinetics. The equilibrium adsorption amount was considered as the final adsorption uptake at the terminal pressure and temperature. N_2_ adsorption isotherms at 25 °C were also recorded using an identical procedure. Ultra high purity (99.9%) grade gas sources were used throughout the study.

## Additional Information

**How to cite this article**: Chowdhury, S. and Balasubramanian, R. Holey graphene frameworks for highly selective post-combustion carbon capture. *Sci. Rep.* **6**, 21537; doi: 10.1038/srep21537 (2016).

## Supplementary Material

Supplementary Information
