# Supplementary material for: Holey graphene frameworks for highly selective post-combustion carbon capture
Source: Sci Rep. 2016 Feb 16;6:21537. doi: 10.1038/srep21537 (PMC4754909; doi:10.1038/srep21537)
Supplement: Supplementary Information [file srep21537-s1.docx]

**Supplementary Information**

**Holey graphene frameworks for highly selective post-combustion carbon capture**

Shamik Chowdhury, Rajasekhar Balasubramanian*

*Department of Civil & Environmental Engineering, National University of Singapore, 1 Engineering Drive 2, Singapore 117576, Republic of Singapore*

**Contents**

**A. Figures**

- **Supplementary Figure 1:** XPS survey scan spectra of HGOs.
- **Supplementary Figure 2:** FTIR spectra of HGFs.
- **Supplementary Figure 3:** FESEM images of (a) HGF-I and (b) HGF-III.
- **Supplementary Figure 4:** Pore size distributions (PSDs) of NGF and HGFs.
- **Supplementary Figure 5:** Surface wettability studies of HGFs.
- **Supplementary Figure 6:** Variation in the CO_2_ adsorption capacity of HGF-II with temperature.
- **Supplementary Figure 7:** CO_2_ adsorption/desorption isotherms of HGF-II at 25 ^o^C.
- **Supplementary Figure 8:** Nonlinear fit of the Toth isotherm model to the experimental CO_2_ equilibrium data of HGF-II.
- **Supplementary Figure 9:** CO_2_ and N_2_ adsorption isotherms of HGF-II as measured at 25 °C.
- **Supplementary Figure 10:** FTIR spectra of virgin and regenerated HGF-II.

**B. Tables**

- **Supplementary Table 1:** Textural properties of the as-prepared NGF and HGFs.
- **Supplementary Table 2:** Comparison of the CO_2_ adsorption capacity of HGF-II with other graphene-based solid adsorbents at 0 ^o^C and 1 bar.
- **Supplementary Table 3:** Toth isotherm parameters for CO_2_ adsorption on HGF-II at different temperatures.
- **Supplementary Table 4:** Comparison of the CO_2_/N_2_ selectivity and purity of the captured CO_2_ for HGF-II with other major types of solid adsorbents at partial pressures relevant to post-combustion carbon capture from the dry flue gas stream of a coal-fired power plant.

**C. Supplementary References**

**Supplementary Figure 1 | XPS survey scan spectra of HGOs.** The O1s peak intensities and atomic ratios (O1s/C1s) of HGOs were significantly decreased in comparison with GO, reflecting the preferential removal of oxygenated carbon atoms and generation of carbon vacancies during sonication with HNO_3_.

**Supplementary Figure 2 | FTIR spectra of HGFs.** The absorption band at 1560 cm^−1^ can be attributed to the C=C skeletal vibration of graphene sheets. The absorption at around 3430 cm^−1^ is due to O−H stretching vibration, implying that a small fraction of hydroxyl and carboxyl functionalities still remained in the HGF samples. The gradual decrease in the O−H band intensity with increasing etchant concentration ascertains that the etching reaction mainly initiates and propagates within the oxygenic defect regions.


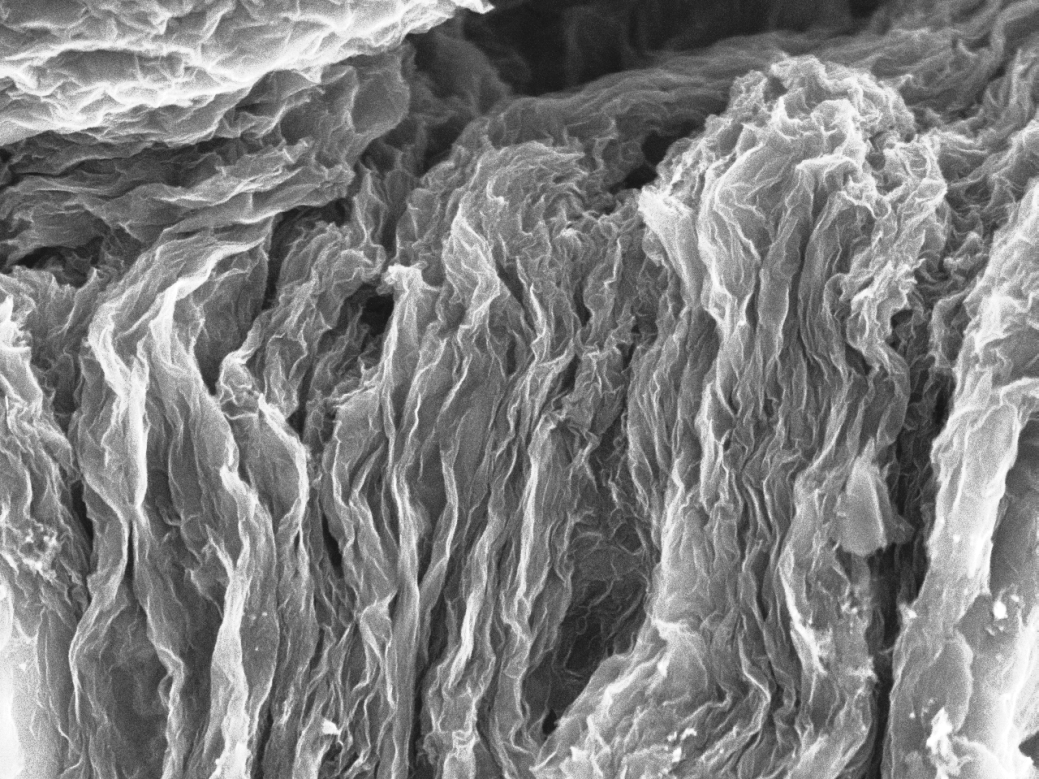

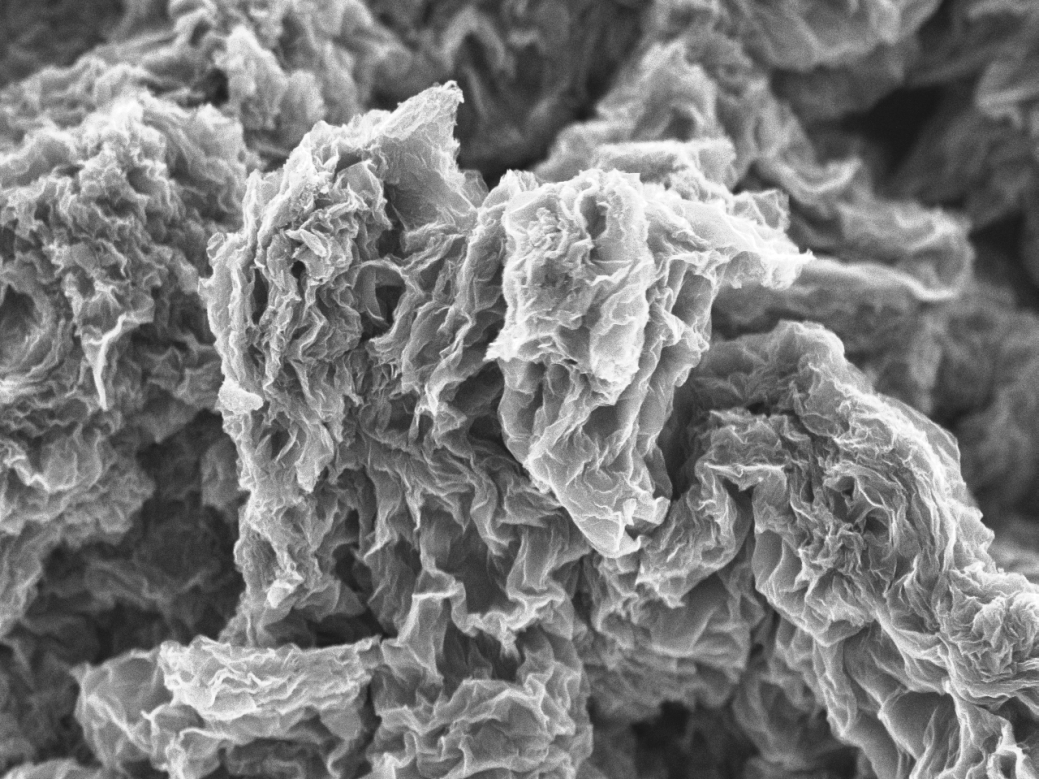


**a**

**b**

**Supplementary Figure 3 |** **FESEM images of (a) HGF-I and (b) HGF-III.** The scale bars represent 1 µm.

**Supplementary Figure 4 |** **Pore size distributions (PSDs) of NGF and HGFs.** The PSD curves were obtained by applying the Barrett–Joyner–Halenda (BJH) method to the desorption branch of the N_2_ isotherms measured at –196 ^o^C.

**Supplementary Figure 5 |** **Surface wettability studies of HGFs.** Top: Illustration of the surface wettability testing of HGFs. Bottom: Dynamic water contact angles of HGFs. The contact angles were greater than 90^o^, indicating that HGFs are hydrophobic.


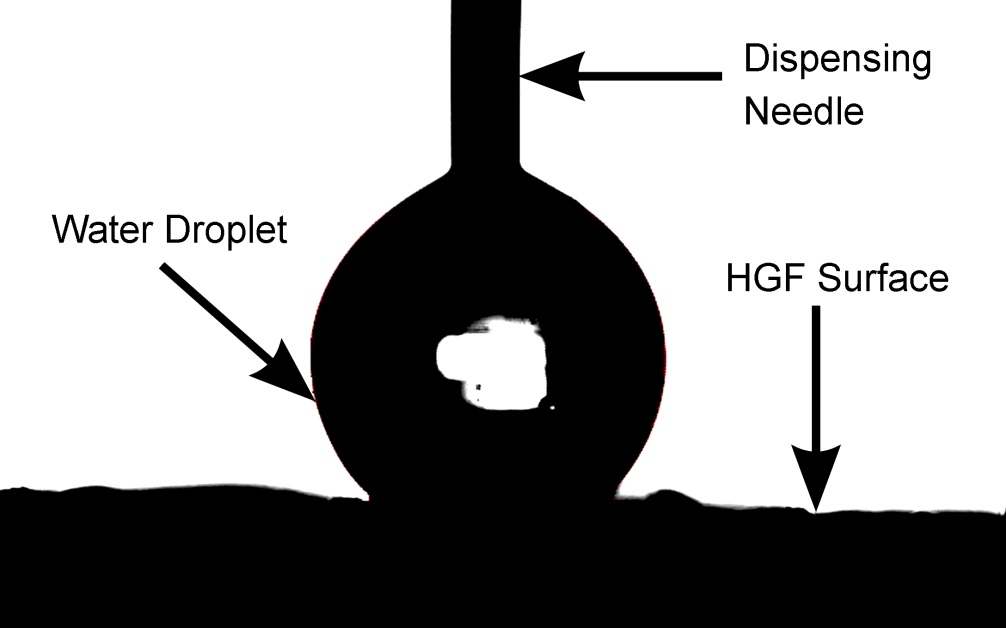


**Supplementary Figure 6 | Variation in the CO_2_ adsorption capacity of HGF-II with temperature.** The observed decrease in adsorption capacity with temperature can be attributed to the exothermic nature of the adsorption process.

**Supplementary Figure 7 | CO_2_ adsorption/desorption isotherms of HGF-II at 25 ^o^C.** The absence of a hysteresis loop indicates that CO_2_ adsorption on HGF-II was completely reversible.

**Supplementary Figure 8 | Nonlinear fit of the Toth (—) isotherm model to the experimental CO_2_ equilibrium data of HGF-II.** The excellent fit of the Toth model over the entire adsorption period suggests that CO_2_ molecules were adsorbed on HGF-II in multimolecular layers.

**Supplementary Figure 9 | CO_2_ and N_2_ adsorption isotherms of HGF-II as measured at 25 °C.** The preferential adsorption of CO_2_ is due to its larger quadrupole moment and higher polarizability than that of N_2_.

**Supplementary Figure 10 | FTIR spectra of virgin and regenerated HGF-II.** The FTIR spectrum of HGF-II after ten repeated cycles of adsorption/desorption shows that there is no change in the framework bonding.

**Supplementary Table 1 |** **Textural properties of the as-prepared NGF and HGFs.** The specific surface area (*S*_sp_) was determined employing the Brunauer–Emmett–Teller (BET) model to the N_2_ adsorption data in the relative pressure (*P*/*P*_0_) range of 0.05–0.20 while the total pore volume (*V*_tot_) was estimated from the amount of N_2_ adsorbed at *P*/*P*_0_ = 0.99. The pore size (*D*_p_) is defined as the size corresponding to the peak maximum in the PSD.

| **Sample** | ***S*_sp_ (m^2^ g^−1^)** | ***V*_tot_ (cm^3^ g^−1^)** | ***D*_p_ (nm)** |
| --- | --- | --- | --- |
| NGF | 198.93 | 0.21 | ─ |
| HGF-I | 439.11 | 1.06 | 3.65 |
| HGF-II | 497.25 | 1.22 | 3.29 |
| HGF-III | 524.18 | 1.27 | 3.74 |

**Supplementary Table 2 |** **Comparison of the CO_2_ adsorption capacity of HGF-II with other graphene-based solid adsorbents at 0 ^o^C and 1 bar.** For a meaningful comparison, the specific surface area and total pore volume of the adsorbents are also given. Clearly, the CO_2_ adsorption in HGF-II is better than or comparable to the other graphene-based materials at similar temperature and pressure conditions. In addition, both the specific surface area and the total pore volume of HGF-II adsorbent is one of the highest among the listed adsorbents.

| **Adsorbent** | ***S*_BET_ (m^2^ g^−1^)** | ***V*_tot_ (cm^3^ g^−1^)*^b^*** | **CO_2_ uptake (mmol g^−1^)** | **Reference** |
| --- | --- | --- | --- | --- |
| 3D Graphene | 477 | 1.0 | 0.7 | Wang *et al*.^1^ |
| Steam activated graphene aerogel | 1230 | 3.67 | 2.45 | Sui *et al*.^2^ |
| GO-based porous carbons | 459 | 1.17 | 1.76 | Xia *et al*.^3^ |
| GO-based hydrogel | 530 | 0.66 | 2.40 | Sui and Han.^4^ |
| Graphene/terpyridine | 440 | 0.34 | 2.65 | Zhou *et al*.^5^ |
| Graphene/Mn_3_O_4_ | 541 | 0.31 | 2.59 | Ding *et al*.^6^ |
| GO/polyethylenimine | 253 ± 22 | 0.7 ± 0.2 | 2.54 | Sui *et al*.^7^ |
| HGF-II | 497 | 1.22 | 2.12 | This study |

**Supplementary Table 3 |** **Toth isotherm parameters for CO_2_ adsorption on HGF-II at different temperatures.** The high *R*^2^ values demonstrate the adequate fit of the Toth model to the experimental equilibrium data over the entire temperature and pressure range.

| ***T*(^o^C)** | | ***q*_s_ (mmol g^-1^)** | | ***b* (bar^-1^)** | | | ***t*** | ***R*^2^** | | |
| --- | --- | --- | --- | --- | --- | --- | --- | --- | --- | --- |
| 0 | | 7.47 | | 5.70 | | | 0.34 | 0.999 | | |
| 25 | | 5.71 | | 1.83 | | | 0.39 | 0.999 | | |
| 50 | | 3.28 | | 1.19 | | | 0.54 | 0.999 | | |
| Temperature dependent Toth isotherm parameters | | | | | | | | | | |
| ***T*_ref_ (K)** | ***q*_s_*,*_0_ (mmol g^−1^)** | | **χ* *** | | ***b*_0_ (bar^−1^)** | **–∆*H*_ads_ (kJ mol^−1^)** | | | ***t*_0_** | **α** |
| 298 | 5.71 | | 3.19 | | 1.83 | 30.78 | | | 0.39 | 0.57 |

**Supplementary Table 4 | Comparison of the CO_2_/N_2_ selectivity and purity of the captured CO_2_ for HGF-II with other major types of solid adsorbents at partial pressures relevant to post-combustion carbon capture from the dry flue gas stream of a coal-fired power plant.** Although HGF-II adsorbs relatively lower amounts of CO_2_ at 0.15 bar than most of the other adsorbents, its CO_2_ over N_2_ adsorption selectivity is the highest, which would indeed be extremely beneficial for extracting a high-purity CO_2_ stream from flue gases for deep underground storage or other industrial applications.

| **Adsorbent** | ***T* (°C)** | **CO_2_ uptake at 0.15 bar (mmol g^−1^)*** | **N_2_ uptake at 0.75 bar (mmol g^−1^)*** | **Selectivity (S_CO2/N2_)**† | **CO_2_ purity (%)**‡ | **Reference** |
| --- | --- | --- | --- | --- | --- | --- |
| *Zeolites* |  |  |  |  |  |  |
| Chabazite | 30 | 0.37 | 0.11 | 16 | 77.08 | Pham *et al*.^8^ |
| K-BEA*^a^* | 25 | 1.16 | 0.22 | 26 | 84.06 | Yang *et al*.^9^ |
| Ca-X*^b^* | 25 | 3.36 | 0.28 | 60 | 92.31 | Bae *et al.*^10^ |
| T-type zeolite nanoparticles | 25 | 2.04 | 0.17 | 59 | 92.31 | Jiang *et al.*^11^ |
| *MOFs* |  |  |  |  |  |  |
| ZIF-8*^c^* | 25 | 0.11 | 0.07 | 8 | 61.11 | McEwen *et al*.^12^ |
| Amino-MIL-53(Al)*^d^* | 25 | 0.92 | 0.19 | 23 | 82.88 | Kim *et al*.^13^ |
| Ni_2_(dobdc)(pip)_0.5_*^e^* | 25 | 1.34 | 0.20 | 33 | 87.01 | Das *et al*.^14^ |
| Bio-MOF-11*^f^* | 25 | 1.22 | 0.09 | 65 | 93.13 | An *et al*.^15^ |
| *Activated carbons* |  |  |  |  |  |  |
| Activated carbon from peanut hull  Activated carbon from sunflower seed shell  Activated carbon from bamboo | 25  25  25 | 1.54  1.46  1.28 | 0.55  0.49  0.41 | 14  15  16 | 73.68  74.87  75.74 | Deng *et al*.^16^  Deng *et al*.^16^  Wei *et al.*^17^ |
| Activated carbon from cellulose fibers | 25 | 1.19 | 0.35 | 17 | 77.27 | Heo and Park.^18^ |
| HGF-II | 25 | 0.53 | 0.03 | 70 | 93.34 | This study |

*^a^* Potassium-exchanged zeolite beta

*^b^* Calcium form of zeolite X

*^c^* Zeolitic imidazolate framework-8

*^d^* Amine functionalized Al(OH)(1,4-benzenedicarboxylate)

*^e^*  Piperazine functionalized Ni_2_(1,4-dioxido-2,5-benzenedicarboxylate)

*^f^* Co_2_(adenine)_2_(CO_2_CH_3_)_2_

* Values estimated from adsorption isotherms in the corresponding reference using WebPlotDigitizer Version 3.8 when not directly reported

† Calculated according to Eq. 1

‡ Calculated according to Eq. 2

**Supplementary References**

1. Wang, Y., Guan, C., Wang, K., Guo, C.X. & Li, C.M. Nitrogen, hydrogen, carbon dioxide, and water vapor sorption properties of three-dimensional graphene. *J. Chem. Eng. Data* **56,** 642-645 (2011).
2. Sui, Z.-Y. *et al*. High surface area porous carbons produced by steam activation of graphene aerogels. *J. Mater. Chem. A* **2,** 9891-9898 (2014).
3. Xia, K., Tian, X., Fei, S. & You, K. Hierarchical porous graphene-based carbons prepared by carbon dioxide activation and their gas adsorption properties. *Int. J. Hydrogen Energy* **39,** 11047-11054 (2014).
4. Sui, Z.-Y. & Han, B.-H. Effect of surface chemistry and textural properties on carbon dioxide uptake in hydrothermally reduced graphene oxide. *Carbon* **82,** 590-598 (2015).
5. Zhou, D. *et al*. Graphene-terpyridine complex hybrid porous material for carbon dioxide adsorption. *Carbon* **66,** 592-598 (2014).
6. Zhou, D. *et al*. Graphene-manganese oxide hybrid porous material and its application in carbon dioxide adsorption. *Chin. Sci. Bull.* **57,** 3059-3064 (2012).
7. Sui, Z.-Y., Cui, Y., Zhu, J.-H. & Han, B.-H. Preparation of three-dimensional graphene oxide-polyethylenimine porous materials as dye and gas adsorbents. *ACS Appl. Mater. Interfaces* **5,** 9172-9179 (2013).
8. Pham, T.D., Xiong, R., Sandler, S.I. & Lobo, R.F. Experimental and computational studies on the adsorption of CO_2_ and N_2_ on pure silica zeolites. *Micropor. Mesopor. Mater.* **185,** 157-166 (2014).
9. Yang, S.-T., Kim, J. & Ahn, W.-S. CO_2_ adsorption over ion-exchanged zeolite beta with alkali and alkaline earth metal ions. *Micropor. Mesopor. Mat.* **135,** 90-94 (2010).
10. Bae, T.-H. *et al*. Evaluation of cation-exchanged zeolite adsorbents for post-combustion carbon dioxide capture. *Energy Environ. Sci.* **6,** 128-138 (2013).
11. Jiang. Q. *et al*. Synthesis of T-type zeolite nanoparticles for the separation of CO_2_/N_2_ and CO_2_/CH_4_ by adsorption process. *Chem. Eng. J.* **230,** 380-388 (2013).
12. McEwen, J., Hayman, J.-D. & Yazaydin, A.O. A comparative study of CO_2_, CH_4_ and N_2_ adsorption in ZIF-8, zeolite-13X and BPL activated carbon. *Chem. Phys.* **412,** 72-76 (2013).
13. Kim, J., Kim, W.Y. & Ahn, W.-S. Amine-functionalized MIL-53(Al) for CO_2_/N_2_ separation: effect of textural properties. *Fuel* **102,** 574-579 (2012).
14. Das, A. *et al*. Carbon dioxide adsorption by physisorption and chemisorption interactions in piperazine-grafted Ni_2_(dobdc) (dobdc = 1,4-dioxido-2,5-benzenedicarboxylate). *Dalton Trans.* **41,** 11739-11744 (2012).
15. An, J., Geib, S.J. & Rosi, N.L. High and selective CO_2_ uptake in a cobalt-adeninate metal-organic framework exhibiting pyrimidine- and amino-decorated pores. *J. Am. Chem. Soc.* **132,** 38-39 (2010).
16. Deng, S. *et al*. Activated carbons prepared from peanut shell and sunflower seed shell for high CO_2_ adsorption. *Adsorption* **21,** 125-133 (2015).
17. Wei, H. *et al*. Granular bamboo-derived activated carbon for high CO_2_ adsorption: the dominant role of narrow micropores. *ChemSusChem* **5,** 2354-2360 (2012).
18. Heo, Y.-J. & Park, S.-J. A role of steam activation on CO_2_ capture and separation of narrow microporous carbons produced from cellulose fibers. *Energy* **91,** 142-150 (2015).
